# MicroRNA profiling associated with non-small cell lung cancer: next generation sequencing detection, experimental validation, and prognostic value

**DOI:** 10.18632/oncotarget.18603

**Published:** 2017-06-22

**Authors:** Sandra Gallach, Eloisa Jantus-Lewintre, Silvia Calabuig-Fariñas, David Montaner, Sergio Alonso, Rafael Sirera, Ana Blasco, Marta Usó, Ricardo Guijarro, Miguel Martorell, Carlos Camps

**Affiliations:** ^1^ Molecular Oncology Laboratory, Fundación Investigación, Hospital General Universitario de Valencia, Valencia, Spain; ^2^ Centro de Investigación Biomédica en Red de Cáncer (CIBEROnc), Madrid, Spain; ^3^ Department of Biotechnology, Universitat Politècnica de València, Valencia, Spain; ^4^ Department of Pathology, Universitat de València, Valencia, Spain; ^5^ Department of Computational Genomics, Centro de Investigación Príncipe Felipe, Valencia, Spain; ^6^ Program of Predictive and Personalized Medicine of Cancer, Institut de Reserca Germans Trias i Pujol (PMPPC-IGTP), Badalona, Spain; ^7^ Department of Medical Oncology, Hospital General Universitario de Valencia, Valencia, Spain; ^8^ Department of Surgery, Universitat de València, Valencia, Spain; ^9^ Department of Thoracic Surgery, Hospital General Universitario de Valencia, Valencia, Spain; ^10^ Department of Pathology, Hospital General Universitario de Valencia, Valencia, Spain; ^11^ Department of Medicine, Universitat de València, Valencia, Spain

**Keywords:** microRNAs, NSCLC, NGS, profiling, prognosis

## Abstract

**Background:**

The average five-year survival for non-small cell lung cancer (NSCLC) patients is approximately 15%. Emerging evidence indicates that microRNAs (miRNAs) constitute a new class of gene regulators in humans that may play an important role in tumorigenesis. Hence, there is growing interest in studying their role as possible new biomarkers whose expression is aberrant in cancer. Therefore, in this study we identified dysregulated miRNAs by next generation sequencing (NGS) and analyzed their prognostic value.

**Methods:**

Sequencing by oligo ligation detection technology was used to identify dysregulated miRNAs in a training cohort comprising paired tumor/normal tissue samples (*N* = 32). We validated 22 randomly selected differentially-expressed miRNAs by quantitative real time PCR in tumor and adjacent normal tissue samples (*N* = 178). Kaplan-Meier survival analysis and Cox regression were used in multivariate analysis to identify independent prognostic biomarkers.

**Results:**

NGS analysis revealed that 39 miRNAs were dysregulated in NSCLC: 28 were upregulated and 11 were downregulated. Twenty-two miRNAs were validated in an independent cohort. Interestingly, the group of patients with high expression of both miRNAs (miR-21^high^ and miR-188^high^) showed shorter relapse-free survival (RFS) and overall survival (OS) times. Multivariate analysis confirmed that this combined signature is an independent prognostic marker for RFS and OS (*p* = 0.001 and *p* < 0.0001, respectively).

**Conclusions:**

NGS technology can specifically identify dysregulated miRNA profiles in resectable NSCLC samples. MiR-21 or miR-188 overexpression correlated with a negative prognosis, and their combined signature may represent a new independent prognostic biomarker for RFS and OS.

## INTRODUCTION

Lung cancer is the leading cause of death from cancer worldwide, with an incidence of more than a million of cases annually [1]. Even though outcomes for patients at all lung cancer stages have improved in recent years, its survival rate remains very poor [2, 3]. Surgery is the only potentially curative treatment for early-stage non-small cell lung cancer (NSCLC) patients. However, there are also many cases that remain uncured following surgery. In fact, 30% to 55% of patients with NSCLC relapse and die from their disease despite curative resection [4, 5]. Hence, there is an urgent need for new biomarkers for this disease which can be used in clinical practice, and accordingly, more studies are also required to identify and validate these new prognostic and diagnostic biomarkers in lung cancer [6–8].

MicroRNAs (miRNAs) are a class of small highly-conserved, non-coding RNAs that were discovered in the early 1990s, and are approximately 18-25 nucleotides long [9–12]. These molecules are important post-transcriptional gene expression regulators related to fundamental processes such as cellular proliferation, differentiation, development, and apoptosis [13]. Altered miRNA levels have been described in several pathologies, including cancer [14–17], and several different studies have suggested that miRNAs could be useful as diagnostic and prognostic biomarkers in lung cancer [18–20]. Additionally, microRNAs have also involved in resistance to chemotherapy and novel targeted agents in non-small cell lung cancer [21]. Furthermore, emerging technologies such as next generation sequencing (NGS) have shown great potential as a platform for small RNA analysis and its use is now being extended to find novel cancer biomarkers.

Therefore, in the context of the above, the aim of this study was to analyze the miRNAome using NGS to characterize dysregulated miRNAs in a large cohort of resectable NSCLC patients, in order to establish expression profiles associated with prognosis in this disease, thus allowing patients with highest risk of relapse to be distinguished.

## RESULTS

The main clinicopathological characteristics of the training (*N* = 32 patients) and validation (*N* = 178 patients) sets including age, gender, stage of disease, and histology are summarized in Table 1. The median follow-up was 81.2 [0.5-110] and 81.2 [1–113] months for training and validation sets, respectively.

**Table 1:** Clinicopathological characteristics of the patients

	Training set		Validation set		*In silico set*	
	N= 32	%	N=178	%	N=618	%
**Age at surgery** (median, range):	64 [47–82]		65 [26–85]		68 [38–88]	
**Gender**						
Male	26	81.2	154	85.5	352	56.9
Female	6	18.8	24	13.5	266	43.1
Stage^(a)^						
I	17	53.1	105	59	350	56.6
II	6	18.8	35	19.7	178	28.8
IIIA	9	28.1	38	21.3	90	14.6
**Histology**						
SCC	13	40.6	84	47.2	279	45.1
ADC	19	59.4	74	41.6	339	54.9
Others			20	11.2		
**Performance Status**						
0	22	68.8	118	66.3	NS	NS
1-2	10	31.2	60	33.7		
**Differentiation grade**						
Poor	10	31.2	43	24.2	NS	NS
Moderate	12	37.5	77	43.2		
Well	7	21.9	31	17.4		
NS	3	9.4	27	15.2		
**Smoking Status**						
Current	16	50	86	48.3	NS	NS
Former	8	25	72	40.5		
Never	8	25	20	11.2		
**Relapse**						
No	20	62.5	98	55.1	373	60.4
Yes	12	37.5	80	44.9	33	5.3
NS					212	34.3
**Dead**						
No	19	59.4	102	57.3	462	74.8
Yes	13	40.6	76	42.7	156	25.2

ADC, adenocarcinoma; SCC, squamous cell carcinoma; NS, non-specified.

^(a)^ According to the American Joint Committee on Cancer staging manual (TNM 6).

### Differential microRNA expression by NGS

Annotation analyses of small RNA biotypes revealed that 88.62% of the small RNAs found were miRNAs. Furthermore, when miRNA annotation was analysed, 940 mature miRNAs had expression in one sample at least. Differential expression analysis was performed to this universe of 940 miRNAs. Analysis of principal components revealed to have two differentiated sample groups in our training cohort corresponding to tumoral and normal samples.

**Figure 1:**
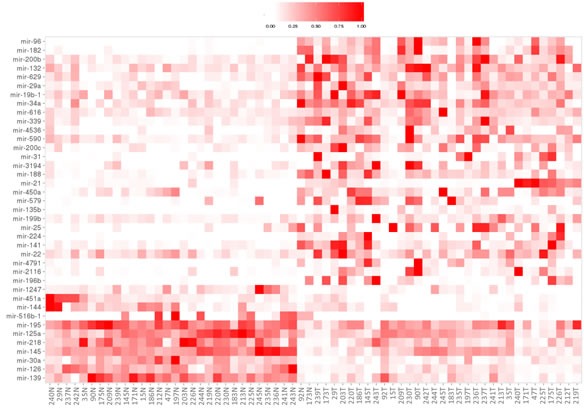
Supervised hierarchical clustering analysis of differentially-expressed miRNAs The level of miRNA expression is color-coded by intensity: white squares represents lower miRNA expression and red squares higher expression in tumor tissue compared with adjacent normal tissue

39 miRNAs were dysregulated in tumor compared to normal samples: 28 were upregulated and 11 downregulated (Table 2). However, differentially-expressed miRNAs were not found when we compared these data with clinicopathological variables. Supervised hierarchical clustering analysis of these differentially-expressed miRNAs revealed two groups of accurately-defined samples (normal and tumor tissues) according to their miRNA expression profile (Figure 1).

**Table 2:** MicroRNAs dysregulated in tumor samples compared to normal samples and Wilcoxon test results for validated microRNAs

A) MicroRNAs upregulated					
	NGS				RTqPCR
Name	stat	FC	*p*-value	p-adj§	*p*-value
miR-96	4.750	5,85	0	0	
miR-182*	3.921	3,70	0	0.009	<0.001
miR-200b	3.882	2,56	0	0.010	
miR-132	3.830	2,08	0	0.011	
miR-629	3.671	2,31	0	0.019	
miR-29a*	3.624	2,30	0	0.020	<0.001
miR-19b-1*	3.615	2,10	0	0.020	<0.001
miR-34a*	3.499	2,20	0	0.026	<0.001
miR-616	3.465	1,83	0.001	0.026	
miR-339*	3.394	2,26	0.001	0.029	<0.001
miR-4536	3.389	4,00	0.001	0.029	
miR-590*	3.376	2,12	0.001	0.029	<0.001
miR-200c	3.375	2,93	0.001	0.029	
miR-31*	3.366	8,99	0.001	0.029	<0.001
miR-3194	3.360	3,45	0.001	0.029	
miR-188*	3.340	2,54	0.001	0.030	<0.001
miR-21*	3.301	4,85	0.001	0.034	<0.001
miR-450a	3.243	2,42	0.001	0.039	
miR-579	3.235	4,37	0.001	0.039	
miR-135b*	3.206	6,63	0.001	0.042	<0.001
miR-199b*	3.180	2,05	0.001	0.043	<0.001
miR-25	3.164	4,34	0.002	0.043	
miR-224	3.160	5,59	0.002	0.043	<0.001
miR-141	3.158	2,72	0.002	0.043	
miR-22	3.153	1,84	0.002	0.043	
miR-4791	3.115	7,24	0.002	0.046	
miR-2116	3.108	4,11	0.002	0.046	
miR-196b*	3.055	7,03	0.002	0.050	<0.001
**B) MicroRNAs downregulated**					
	**NGS**				**RTqPCR**
**Name**	**stat**	**FC**	***p*-value**	**p-adj§**	***p*-value**
miR-1247	−3.144	−2,89	0.002	0.043	
miR-451a*	−3.466	−3,43	0.001	0.026	<0.001
miR-144*	−3.494	−2,84	0	0.026	<0.001
miR-516b-1	−3.535	−43,319	0	0.025	
miR-195*	−3.927	−1,57	0	0.009	0.039
miR-125a*	−4.392	−1,78	0	0.002	0.679
miR-218*	−4.467	−2,06	0	0.001	<0.001
miR-145*	−4.778	−1,89	0	0	0.001
miR-30a*	−5.141	−2,81	0	0	<0.001
miR-126*	−5.795	−2,75	0	0	<0.001
miR-139*	−6.477	−2,93	0	0	<0.001

A) MicroRNAs upregulated and B) MicroRNAs downregulated.

FC, fold change

§ p-adj calculated with FDR method

*miRNAs validated by RTqPCR.

### Functional analyses

*In silico* functional studies, based on computational analyses from 39 miRNA found dysregulated in this study, showed several biological processes (bp) of GO terms significantly related to lung, such as, respiratory system development, lung development, respiratory tube development, which seems to indicate that these differentially expressed miRNAs could be tissue specific. Furthermore, response to growth factor stimulus and cellular response to growth factor stimulus GO terms were found enriched in this analysis. This fact is in concordance with the increase in cellular growth during carcinogenesis (Table 3). In addition, functional analyses were performed with the prognostic value miRNAs (miR-21 and miR-188), where target gene enrichments were carry out. Analyses showed an elevated number of target genes for both miRNAs related to essential pathways in carcinogenesis process (Supplemental Figure 1).

**Table 3:** Gene-Ontology terms dysregulated by differentially expressed miRNA

	N	lor	pval	padj	pat	GO
GO:0007517	306	0,258453	9,59E-06	0,031571	1	muscle organ development
GO:0060541	151	0,34596	2,75E-05	0,031571	1	respiratory system development
GO:0070848	498	0,191793	3,24E-05	0,031571	1	response to growth factor stimulus
GO:0071363	489	0,1885	5,12E-05	0,031571	1	cellular response to growth factor stimulus
GO:0030324	131	0,358327	5,18E-05	0,031571	1	lung development
GO:0008543	150	0,336667	5,42E-05	0,031571	1	fibroblast growth factor receptor signaling pathway
GO:0061061	406	0,204676	5,69E-05	0,031571	1	muscle structure development
GO:0030323	134	0,348344	6,87E-05	0,033321	1	respiratory tube development
GO:0050767	336	0,215493	0,000109	0,040653	1	regulation of neurogenesis
GO:0048015	148	0,320334	0,000126	0,040653	1	phosphatidylinositol-mediated signaling
GO:0048017	148	0,320334	0,000126	0,040653	1	inositol lipid-mediated signaling
GO:0044087	296	0,226529	0,000128	0,040653	1	regulation of cellular component biogenesis
GO:1901698	468	0,180575	0,000137	0,040653	1	response to nitrogen compound
GO:0010243	441	0,184938	0,000147	0,040653	1	response to organonitrogen compound
GO:0035295	381	0,197236	0,000164	0,042486	1	tube development
GO:0030510	56	0,502461	0,000201	0,045892	1	regulation of BMP signaling pathway
GO:0033238	42	−0,55751	0,000193	0,045892	−1	regulation of cellular amine metabolic process

N, number of genes; lor, Log Odds Ratio; pval, p-value; padj, p-value adjusted by multiple test method; pat, pathway; GO, Gene onthology term (biological process).

### MicroRNA expression validation

From the 39 differentially-expressed miRNAs, seven presented an average of fewer than 30 counts and so were excluded from the validation analysis. Twenty-two miRNAs were randomly selected for validation in an independent cohort of patients (*N* = 178). The Wilcoxon signed-rank test, confirmed that there were statistically significant differences in the expression of these miRNAs between tumor and normal adjacent lung tissue with the exception of miR-125a (Table 2A-2B).

### Prognostic value of microRNAs

**Figure 2:**
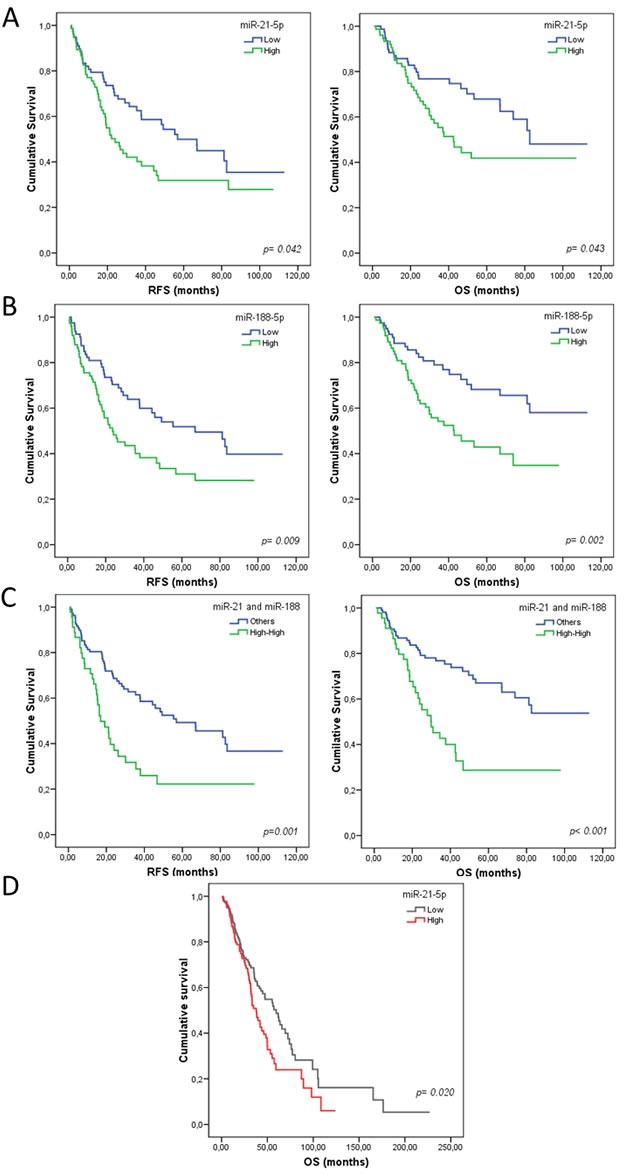
Kaplan-Meier curves for overall survival (OS) and relapse-free survival (RFS) according to miRNA expression for patients with resected non-small cell lung cancer (NSCLC) Panels **A** and **B** show OS and RFS for miR-21-5p and miR-188-5p, respectively in the validation set. Panel **C** shows the significant correlation between the combined variable, high expression of both miRNAs (miR-21high and miR-188high), with shorter OS and RFS times in validation set. Panel **D** shows OS and RFS for miR-21 in the *in silico* set. Continuous variables were dichotomized as high (≥ median) and low (< median), using the median relative expression of each gene as a cutoff. Statistics were calculated using the log-rank test, and the significance was set at *p* < 0.05.

Of the 22 miRNAs analyzed, only miR-21-5p and miR-188-5p had any prognostic value: higher expression of these miRNAs was significantly correlated with shorter RFS (24.03 *vs*. 56.83 months, *p* = 0.042 and 23.67 *vs*. 66.97 months, *p* = 0.009, respectively) and OS (42.60 *vs*. 82.60 months, *p* = 0.043 and 42.90 *vs*. 81.23 months, *p* = 0.002, respectively) (Table 4, Figure 2A-2B). According to these results, and in order to better assess the prognosis of patients, we also considered the combination of these two microRNAs. Interestingly, patients with high expression of both miRNAs (miR-21^high^ and miR-188^high^) had shorter RFS and OS times (*p* = 0.006 and *p* = 0.0006, respectively) (Table 4, Figure 2C).

**Table 4:** Univariate (log-rank test) and multivariate (Cox regression model) analyses for RFS and OS

	Univariate				Multivariate					
	RFS		OS		RFS			OS		
Variable	Median (months)	*p*^§^	Median (months)	*p*^§^	HR	95% CI	*p*	HR	95% CI	*p*
**miR-21-5p** (high vs. low)	24.03 vs. 56.83	0.042	42.60 vs. 82.60	0.043	--	--	--	--	--	--
**miR-188-5p** (high vs. low)	23.67 vs. 66.97	0.009	42.90 vs. NR	0.002	--	--	--	--	--	--
**Combined miRNAs** (miR-21^high^ miR-188^high^ vs. other combinations)	16.97 vs. 56.83	0.001 (0.006)*	29.90 vs. NR	< 0.0001 (< 0.0006)*	2.170	1.372-3.431	0.001	3.256	1.907-5.561	<0.0001
**KRAS Status** (mutated vs. WT)	16.97 vs. 46.67	0.034	27.90 vs. NR	0.038	2.076	1.215-3.831	0.020			
**Lymph node involvement** (yes vs. no)	26.23 vs. 48.33	0.029	--	--	--	--	--	--	--	--

§ *p*-value was calculated using the log-rank test. * *p*-values with Bonferroni corrections are shown in parentheses. CI, confidence interval; HR, hazard ratio; NR, not-reached; OS, overall survival; RFS, relapse-free survival.

### Multivariate Cox regression analysis

To determine which analyzed variables had an independent impact on the lung cancer prognosis in our cohort, we performed multivariate analysis. The Clinical and experimental variables included were all those that were statistically significant in the univariate analysis. *KRAS* status was an independent prognostic variable for RFS according to the Cox regression model (*p* = 0.020) and the signature (miR-21^high^ and miR-188^high^) was an independent poor prognostic biomarker for RFS (*p* = 0.001) and OS (*p* < 0.0001) (Table 4).

### Prognostic signature validation

Data from TCGA for squamous lung cancer (LUSC) and lung adenocarcinoma (LUAD) patients was used for *in silico* validation of the miRNA signature. Clinicopathological characteristics of these patients are summarized in Table 1. The analyses confirmed that both miR-21 and miR-188 were significantly overexpressed in tumor tissues (*p* < 0.0001). Patients with post-surgical complications were excluded from the survival analysis, and only those patients who had at least 1 month of follow up were included (*N* = 496). Analysis revealed a poor prognosis for OS in patients with high levels of miR-21 (37.86 *vs* 59.66 months, *p* = 0.020) (Figure 2D); however miR-188 did not show prognostic value in the group of TCGA patients. RFS analysis was not carried out as the data for disease status was not specified in many of the patients.

## DISCUSSION

MicroRNAs have been shown to play an important role in the tumorigenesis and development of NSCLC. However, few studies characterize the profiles of miRNAs in paired tumor and adjacent normal tissue samples at the same time. In our study, the microRNAome was assessed in a large cohort (training set = 32 and validation set = 178, respectively) of resectable NSCLC patients, who long-term follow-ups were available, thus strengthening the analysis. Additionally, this study presents the useful application of deep next generation sequencing technology for profiling miRNAs in resectable lung tumor specimens. Some similar studies have been performed using different microarray platforms [22, 23], ours being one of the first to analyze dysregulated miRNAs in fresh frozen tumor and adjacent normal samples of a large cohort of characterized NSCLC patients using SOLiD technology.

NGS technology allows the whole microRNAome of each sample to be analyzed with high sensitivity, a wide dynamic range, high accuracy, and extraordinary technical reproducibility [24, 25]. In the cancer research context, NGS technology is especially useful compared to other methodologies because it allows the detection of miRNAs not yet described [24, 26, 27]. However, there are some limitations in using NGS, including its high cost and the large amounts of data it produces. Nonetheless, the development of new platforms using barcodes, are now becoming available. This allow several samples to be multiplexed into a single run [28], and is contributing to a continuing cost reduction and new bioinformatics tools for analyzing and interpreting these complex data sets [27, 29].

Of the 39 dysregulated miRNAs in the training set, 22 miRNAs were validated using RTqPCR, which is considered one of the gold standard techniques for gene expression analyses due to its sensitivity and robustness requiring minimal amounts of RNA [30–32]. Functional analyses of these dysregulated miRNAs shown an enrichment of several biological processes related to lung specifically, such as, respiratory system development, lung development, respiratory tube development, which seem to indicate that these differentially expressed miRNAs could be tissue specific. Interestingly, other biological processes, related to cellular response to growth factor stimulus were found enriched in this analysis as well. This fact is in concordance with an increased growth cell in carcinogenesis process.

Regarding the analyzed miRNAs, the overexpression of miR-182, miR-31, miR135b, miR-199b, miR-224 and miR-196b and miR-34a have been detected in both the training and validation set. Extensive profiling studies have connected dysregulated levels of miR-182 with several cancer types, including NSCLC [33]. Furthermore, miR-182 may function as an oncogenic miRNA to enhance cancer cell proliferation, survival, aggressiveness, tumorigenesis, and drug resistance [34–36]. Some targets of miR-182 are involved in repaired DNA [37] and others are tumor suppressor genes such as *PTEN* and *TP53* (miRTarBase data). A meta-analysis in various cancers has shown the overexpression of miR-31 [38], which was overexpressed in early stages, and expression was high in tumor progression and reached higher levels in advanced stages. MiR-31 has been shown to act as an oncogenic miRNA by targeting specific tumor suppressors, including the large tumor suppressor 2 (*LATS2*) and PP2A regulatory subunit B alpha isoform (*PPP2R2A*) [39]. An analysis of the predicted targets of miR-31 found a relationship between this miRNAs and the initiation, progression and treatment response of lung cancer through the cell cycle, the cytochrome P450 pathway, metabolic pathways, apoptosis, the chemokine signaling pathway, and the MAPK signaling pathway [40].

A few studies have described miR-135b and its relationship in NSCLC, but only Lin et al. have found upregulated miR-135b in invasive NSCLC cells [41]. Other studies in different cancer types have found an overexpression in miR-135b [42, 43]. MiR-135b expression enhances cancer cell invasive and migratory abilities *in vitro* and promotes cancer metastasis *in vivo* by targeting multiple key components on the Hippo pathway, including *LATS2*, *BTRCP* and *NDR2*, as well as *LZTS1* [41]. Regarding miR-224, some studies in the same type [44] and different types of cancer support our results [45, 46]. MiR-224 functions as an oncogene in NSCLC by directly targeting *TNFAIP1* and *SMAD4*. Caspase 3 (*CASP3*) and caspase 7 (*CASP7*) are targets of miR-224 in NSCLC, and miR-224 partly promotes lung cancer cells proliferation and migration by directly targeting *CASP7* and downregulating its expression; miR-224 attenuates *TNF-α* induced apoptosis by directly targeting *CASP3*, which results in a reduced cleaved parp1 expression in lung cancer cells and suggests a oncogenic role for miR-224 in lung cancer pathogenesis [47]. Two studies in lung cancer tissues and lung adenocarcinomas support our data about miR-196b [48, 49]. The same results have been found in different cancer types [50]. *HOXA9* has been suggested to act as a target of miR-196b by playing a central role in controlling the aggressive behavior of lung cancer cells [49]. Unexpectedly, miR-34a was found upregulated in the training and validation set. MiR-34a has been reported to be down-regulated in NSCLC [51] other cancer types and to work as tumor suppressor miRNA. Considering all these results, more studies need to be performed in more extended cohorts of patients to be able to better understand the function of miR-34a in NSCLC.

The down-regulation of miR-451a, miR-144, miR-195, miR-218, miR-145, miR-30a, miR-126 and miR-139 has been found in both the training and validation set. Two meta-analyses in NSCLC have demonstrated the reduced expression of these eight miRNAs in tumor [52, 53]. *AKT* and oncogene *MYC* have been described as targets, and a role of miR-451a in the carcinogenesis process has been shown [54]. The met proto-oncogene (*MET*), which is often amplified in human cancers and functions as an important regulator of cell growth and tumor invasion, has been identified as a direct target of miR-144 [55]. MiR-195 overexpression inhibits ACHN cell viability, migration, and invasion, and also induces cell apoptosis by targeting *VEGFR2* *via* the PI3K/AKT and Raf/MEK/ERK signaling pathways, which indicates that miR-195 plays a tumor suppressive role [56]. Studies *in vitro* have shown an anti-cancer function of miR-218, and the down-expression of miR-218 increases myocyte enhancer factor 2D (*MEF2D*) levels by promoting lung cancer growth [57], and by inhibiting NSCLC proliferation and metastasis *via* by downregulating *CDCP1* [58]. The down-regulation of miR-218 increases epithelial-mesenchymal transition and tumor metastasis in lung cancer by targeting Slug/ZEB2 signaling [59]. *In vitro* analyses have shown a tumor suppressor role of miR-145, where miR-145 inhibits *TGF-β*-induced epithelial-mesenchymal transition and invasion through the repression of *SMAD3* and *TGFBR2* in NSCLC cells [60, 61]. Besides, miR-145 inhibits the migration and invasion of lung cancer cells *via* fascin homolog 1 (*FSCN1*) downregulation [62] and cell growth is inhibited by miR-145, while *MYC* has been shown to be a direct target for miR-145 [63]. MiR-30a overexpression in lung tumor culture cells inhibits migration and cell invasion, and partially attributes to lower EYA2 expression [64] and influences NSCLC progression through the PI3K/AKT signaling pathway by targeting *IGF1R* [65]. MiR-126 is known to play a critical role in the angiogenesis process [17, 66]. Furthermore *in vitro* analyses have shown that miR-126 overexpression in the NSCLC line cell inhibits cell growth through different gene targets, including *CRK*, *EGFL7* and *PIK3R2* [67–69]. Dysregulated miR-139 expression has been reported in some human tumor types. Analyses *in vitro* and *in vivo* have demonstrated miR-139-5p suppressed tumor growth and directly targeted *MET*, which could be a possible mechanism by which miR-139-5p regulates growth and the metastatic potential [70].

Other miRNAs such as miR-29a, miR-199-5p, miR-339-5p, miR-590-5p and miR-19b-1 are overexpressed in both the training and validation set, but have barely been described in cancer in general and in NSCLC in particular. Only one study in lung adenocarcinoma has identified miR-29a overexpression in a paired-sample analysis (tumor and normal from the same patients) [71]. Regarding miR-199-5p, only one study in lung cancer and miRNA changes related to asbestos has reported the same results [72]. MiR-339-5p overexpression has been reported in a study performed in the peripheral blood of 100 individuals which included 86 patients with predominantly early-stage NSCLC and 24 healthy donors [73]. MiR-590-5p overexpression has not been reported in NSCLC, but studies support our findings in other cancer types [74, 75]. Although miR-19b-1 is not widely described in the literature, miR-19b overexpression was validated in our validation cohort. To support this finding, miR-19b-1 upregulation has been observed in HeLa human cancer cells and in mice lung tumors [76], but no similar study to ours is currently available which detects deregulation in this miRNA. For this reason, more studies are needed to better understand the relationship between these miRNAs and the carcinogenesis process of NSCLC.

In agreement with our findings, miR-21 has been reported to be overexpressed in several solid malignancies [77–79], including lung cancer [80, 81] where several studies in paired tumor and adjacent normal tissue samples in NSCLC showed similar results [18, 71, 82]. Interestingly, different meta-analysis studies performed in many tumor types [83, 84] and which only considered NSCLC [52, 53] confirm this finding. Therefore, *in silico* analyses have shown that miR-21 has large number of genes targets and many of them are directly involved in essential pathways dysregulated in the tumorigenesis process.

The analysis of the prognostic value of dysregulated miRNAs show that higher relative expression of miR-21 and miR-188 is associated with worse outcome in our cohort of resectable NSCLC. These data agreed with the *in silico* study performed on data obtained from the TCGA cohort, confirming the overexpression of both miRNAs in tumor samples compared to normal lung tissues. In the TCGA set, patients with higher expression of miR-21 exhibited shorter OS (37.86 *vs* 59.66 months, *p* = 0.020). In line with these results, some studies have reported that high levels of miR-21 in either tumor or blood samples were associated with a negative prognosis in lung cancer patients [15, 83, 85, 86]. Interestingly, Seike et al. described that aberrant miR-21 expression; enhanced by the activated EGFR signaling pathway, played a significant role in lung carcinogenesis in those that never-smoked [82]. Mechanistically, it has been described that overexpression of miR-21 leads to the inhibition of several tumor suppressor genes, such as *PTEN*, *TPM1* and *PDCD4* [87]. Thus, miR-21 seems to be a promising biomarker and is also an interesting therapeutic target whose inhibition may have benefits in NSCLC patients. Recently, a preclinical study in a murine lung cancer model with an anti-miR-21 molecule revealed that treated animals displayed tumor regression or no tumor growth and prolonged survival while compared with the untreated group [88].

Our results obtained in the validation set by RTqPCR also indicated that patients with elevated miR-188 expression in tumor tissue had a shorter RFS (*p* = 0.009) and OS (*p* = 0.002). However, these findings could not be confirmed by *in silico* analysis in the TCGA cohort due to probably technical (pair tumor/normal analysis, NGS *vs* RTqPCR) and biological (amount of miRNA) differences. Several interpretations can explain this results. Initially, miRNA data expression of patients in our validation cohort was analyzed using RTqPCR while the TCGA validation cohort samples were analyzed by NGS. The different sensitivity between these two techniques could explain, in part, these different results. Otherwise, a distinct concept to calculate relative expression could have influence over them too. In validation set, the relative miRNA expression was calculated as the number of times the tumor tissue expression was higher or lower compared to its paired-normal tissue for each patient (fold-change) whereas the relative expression of TCGA data was calculated by RPKM. Despite the miRNA profiling techniques have vastly improved in the last years, there are still differences in performance and platform-specific biases that can impact the generated output [28]. Finally, the expression levels of miR-21 were high in normal and tumor samples, due to its constitutive expression, which is frequently upregulated in solid tumours. Therefore, the range RPKM values between high and low normalized was wide, so when dichotomization is performed, patients with higher or lower values from median are clearly defined and Kaplan-Meier analysis shown two well differentiated and significative populations of patients with a clearly different prognosis. In contrast, the expression levels of miR-188 were very low in normal and even in tumor samples. Consequently, the ranges between normalized values were narrow, so when dichotomization is performed, patients with higher or lower values from median were not clearly differentiated and Kaplan-Meier differences found were not significative. This can explain why miR-188 cannot find a prognosis value in TCGA cohort.

There are limited studies analyzing the prognostic role of miR-188 in cancer [89–91], and in fact none has been found in lung cancer to date. In concordance with our results, in patients with acute myeloid leukemia (cytogenetically-normal), high miR-188 expression has been significantly associated with shorter OS and event-free survival [89]. Although the computational analysis has shown a relationship of miR-188 with the carcinogenesis process, further studies are still needed to clarify the function of this miRNA in carcinogenesis and its prognostic implication in cancer.

Finally, we identified a patient subgroup defined by a combined higher expression levels of miR-21 and miR-188 that exhibited poor outcome and resulted in an independent prognostic factor for RFS (HR: 0.485 [0.313-0.753]; *p* = 0.001) and OS (HR 0.389 [0.237-0.638]; *p* < 0.0001) in multivariate analysis, suggesting its potential role as biomarker useful for distinguishing patients which could benefit from more exhaustive monitoring.

In summary, the miRNA signature identified in this work may provide new biomarkers for providing early prognoses in NSCLC patients as well as being potentially useful as a therapeutic target for this disease in the near future.

## CONCLUSIONS

This study revealed that the NGS system can accurately detect miRNAs and specifically identify dysregulated miRNAs in resectable NSCLC samples; furthermore, these results were validated in a large independent cohort of patients. Survival analyses showed that miR-21-5p or miR-188-5p overexpression were negative prognostic factors, implicating miR-188 in NSCLC for the first time. Furthermore, the combined signature of these two miRNAs was significantly associated with shorter RFS and OS times and was confirmed by a multivariate analysis as an independent prognostic marker, representing a potential novel negative prognostic biomarker for NSCLC. However, especially considering that the role of miR-188 in NSCLC remains unclear, further studies must be performed in diverse populations and using functional evaluation methods in order to confirm and extend our findings. Given that, to date, very few studies have used paired fresh frozen-tissues, and it would be interesting to extend this study in NSCLC, using a greater number of samples, as well as in other tumor types. We consider our findings to be important in both translational clinical research and in the development of novel miRNA-based cancer therapies.

## MATERIALS AND METHODS

### Patients and tissue samples

This retrospective study included 32 patients in the training set, and 178 patients in the validation set with NSCLC from the *Consorcio Hospital General Universitario de Valencia* who underwent surgery between 2004 and 2013, and who fit the eligibility criteria: resected, non-pretreated stage I to IIIA patients (according to the American Joint Committee on Cancer staging manual) with a histological diagnosis of NSCLC. Tumor and adjacent normal lung specimens were obtained by surgical resection and were preserved in RNA-later at −80°C until analysis (Applied Biosystems, USA). The study was conducted in accordance with the Declaration of Helsinki, and the institutional ethical review board approved the protocol. Relapse-free survival (RFS) was estimated as the time from surgery to recurrence or death from the disease, whereas overall survival (OS) was defined as the time from diagnosis to the date of death or the patient's last follow-up. *In silico* validation set included data from 618 resectable NSCLC caucasian patients from the TCGA project.

### RNA isolation and quality evaluation

Total RNA isolation from the tumor and normal fresh frozen tissues was performed using TRI Reagent (Sigma, USA) according to the manufacturer's instructions. RNA samples were subjected to quality control before carrying out NGS; using RNA Nano and Small RNA Chips on an Agilent 2100 Bioanalyzer (Agilent technologies, Germany). The miRNA fraction was enriched with an optimal profile using a PureLink miRNA isolation kit (Invitrogen, USA). After performing quality control, 32 of the tested samples had an optimal quality profile for NGS sequencing.

### NGS

Small RNA sequencing using a sample barcode-identification multiplex with SOLiD (Sequencing by Oligo Ligation Detection) technology was performed. A SOLiD total RNA-seq kit was used to prepare small RNA libraries and templated beads were subsequently prepared according to the manufacturer's instructions. Briefly, the libraries were constructed and the cDNAs amplified with the 3′ PCR primers supplied in the SOLiD RNA barcoding kit containing the P2 sequence required for emulsion PCR (ePCR) using beads. After ePCR and bead enrichment, the samples were deposited onto a slide and sequenced on the SOLiD^TM^ 4 System (Applied Biosystems, USA).

### RTqPCR

The expression of 22 randomly-selected miRNAs was validated in an independent cohort using miRNA-specific TaqMan assays (Applied Biosystems). Briefly, reverse transcription (RT) was performed with 500 ng of total RNA using a TaqMan MicroRNA reverse transcription kit (Applied Biosystems) following the multiplex RT protocol, according to the manufacturer's instructions. Normalization and relative expression quantification was calculated with miR-16 using the comparative Cq method (2^−(Cq sample-Cq control)^) to validate the miRNA expression and used the Pfaffl formula to perform survival analysis.

### Bioinformatic analysis

In the training set, color space fasta (csfasta) and quality (qual) format input data were grouped for each sample and transformed into FASTQ files in order to import them into the CLC Genomics Workbench software (version 5.5.2; CLC bio, Denmark). The trimming and count were performed together to remove adapter sequences and to count different tags. The miRNAs were subsequently annotated using the miRBase platform (release 20.0, *Homo sapiens*) and the Ensembl.org or tRNAscan-SE databases for other small RNA biotypes. Reads for the same mature miRNAs were grouped and normalized by totals, reads per kilobase per million mapped reads (RPKM). Principal components analysis was performed to visualize the sample grouping and the Baggerley test was used to analyze differential miRNA expression between tumor and normal tissues and the FDR (false discovery rate) was used to adjust *p*-values.

Functional analyses were carried out using a new bioinformatics algorithm [92] with differentially dysregulated miRNAs, applying statistics and p-values obtained from Baggerley test.

*In silico* validation was performed using two lung cancer data sets from the TCGA consortium [93, 94]. Clinical and miRNA-seq (Illumina HiSeq platform) information was directly downloaded from the ICGC Data Portal [95], https://dcc.icgc.org/releases/current/projects/LUAD-US, and https://dcc.icgc.org/releases/current/projects/LUSC-US MiR-21-5p and miR-188-5p RPKM values were extracted using their genomic positions, obtained from miRBase. *T*-tests paired (p1, only cases with paired (p1, only cases with paired tumor and normal samples, *N* = 71) and non-paired (p2, comparison of all tumor samples vs all normal samples) were carried out on log-transformed expression values to analyse differential expression, using R statistical environment [96]. Analyses were considered to be statistically significant to *p*-value ≤ 0.05.

### Statistical analysis

Mann-Whitney and Chi-square tests were applied to confirm that there were not statistically significant differences in clinicopathological characteristics between both, training and validation sets. The Wilcoxon matched-pairs test was used to validate NGS results tested by RTqPCR. The survival curves were plotted according to the univariate Kaplan-Meier method (log-rank) with clinicopathological variables and dichotomized microRNA expression levels. For multiple comparisons, the Bonferroni method was applied, maintaining the overall probability of *ƙp* < 0.05. Finally, a Cox model for multivariate analyses was used to assess the independent value of the tested biomarkers. Statistical analysis was performed using the Statistical Package for the Social Sciences (SPSS, USA) version 15.0. A *p*-value ≤ 0.05 was considered to be statistically significant for all analyses.

## SUPPLEMENTARY MATERIALS FIGURE


