# Supplementary material for: MicroRNA profiling associated with non-small cell lung cancer: next generation sequencing detection, experimental validation, and prognostic value
Source: Oncotarget. 2017 Jun 22;8(34):56143–57. doi: 10.18632/oncotarget.18603 (PMC5593550; doi:10.18632/oncotarget.18603)
Supplement: Supplementary file 1 [file oncotarget-08-56143-s001.pdf]

MicroRNA profiling associated with non-small cell lung cancer: next generation sequencing detection, experimental validation, and prognostic value

SUPPLEMENTARY MATERIALS

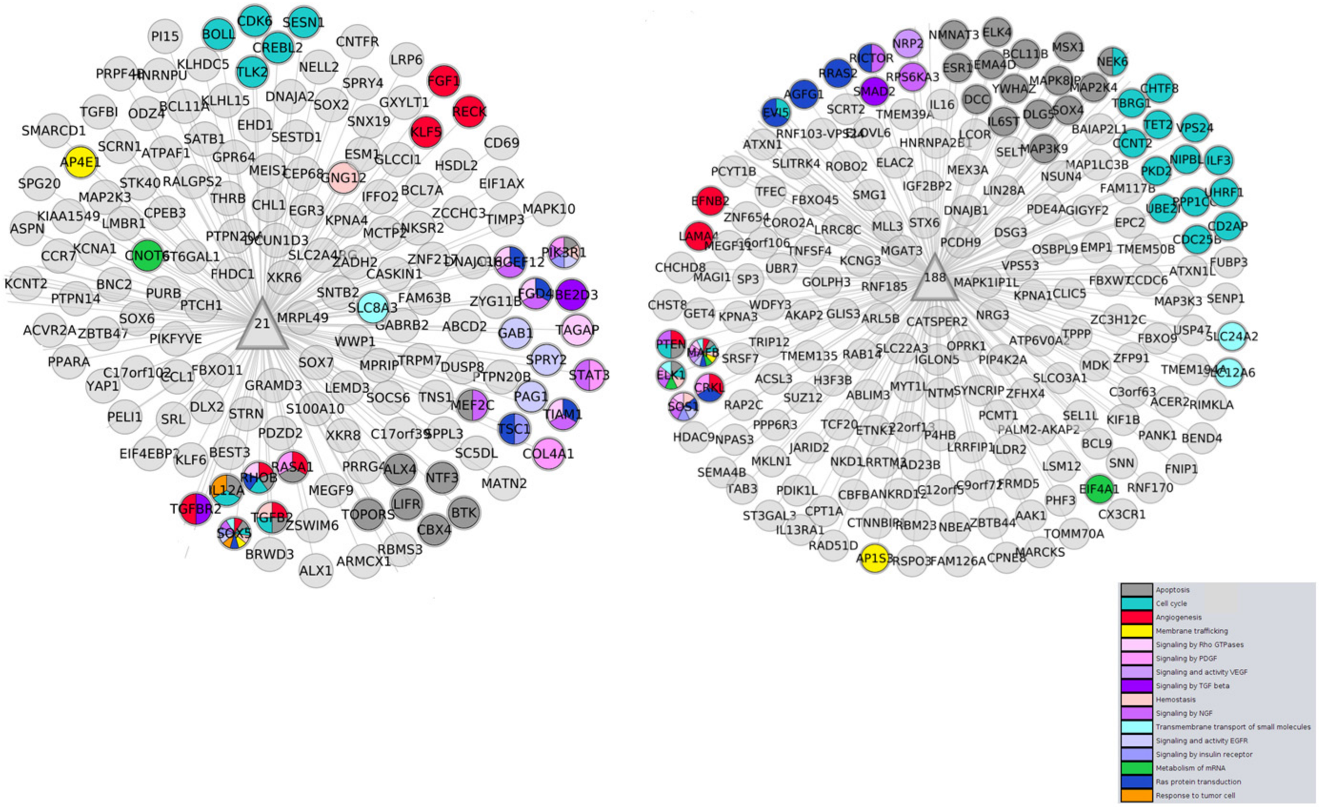

Supplementary Figure 1: Target genes enrichment for miR-21 and miR-188
